# KH550 and Sodium Palmitate Mediated Synergistic Modification of Micronized Silver Powder: Insights into Dispersion Regulation and TOPCon Paste Performance Optimization

**DOI:** 10.3390/ma19183939

**Published:** 2026-09-16

**Authors:** Jun Yin, Ruxiao Jia, Kejun Zhong, Lei Zhang, Jia Liu, Lianzhou Zhang, Hao Sun, Wenyan Zhang, Ying Zhao, Mi Zhang

**Affiliations:** 1 Key Laboratory of Synthetic and Natural Functional Molecule of the Ministry of Education, College of Chemistry & Materials Science, Northwest University, Xi’an 710127, China,; 2 Emperor Qinshihuang’s Mausoleum Site Museum, Xi’an 710600, China; 3 Xi’an Huichuang Precious Metal New Materials Research Institute Co., Ltd., Xi’an 710300, China; 4 Xi’an Hongxing Electronic Paste Technology Co., Ltd., Xi’an 710199, China; 5 Shaanxi Provincial Institute of Basic Sciences (Chemistry & Biology), Northwest University, Xi’an 710127, China

**Keywords:** micron-sized silver powder, KH550, sodium palmitate, molecular synergy, interfacial bonding

## Abstract

**Highlights:**

A novel synergistic modification strategy using KH550 and sodium palmitate is proposed to address agglomeration and compatibility issues of micronized silver powder in TOPCon cells.

**What are the main findings?**

- A novel KH550 and sodium palmitate synergistic modification strategy is proposed for micron silver powder in TOPCon cells.
- Modified silver powder exhibits excellent dispersibility with 6.01 g/cm^3^ tapped density and a contact angle of 141.5°.
- The corresponding silver paste achieves optimized performance with reduced viscosity, low resistivity and a 45.18% aspect ratio.

**What are the implications of the main findings?**

- This approach enables precise control over the dispersion and printability of silver powder.
- It provides a strong theoretical foundation for the design of various silver paste carrier systems.

**Abstract:**

Micron-sized silver powder plays a crucial role in Tunnel Oxide Passivated Contact solar cells (TOPCon) within the photovoltaic field due to its high bulk density and excellent rheological properties. Considering that micron-sized silver powder produced by the liquid-phase reduction method exhibits characteristics such as high surface energy and a tendency to agglomerate, along with poor compatibility with organic carriers in the paste, this work employs a strategy of synergistically modifying micron-sized silver powder with KH550 and sodium palmitate to regulate silver powder dispersion and paste adaptability. The composite-modified silver powder exhibits an increase in tapped density from 4.25 g/cm^3^ to 6.01 g/cm^3^, a bulk density elevated from 2.98 g/cm^3^ to 4.11 g/cm^3^, and a contact angle increased from 46° to 141.5°, demonstrating significantly enhanced dispersion. The TOPCon silver paste prepared from the modified silver powder exhibited excellent comprehensive properties: a viscosity of 6.43 × 10^6^ cp, a low resistivity of 2.75 × 10^−6^ Ω·cm, and a high aspect ratio of 45.18%. This work elucidates the impact of surface multifunctionalization on silver powder dispersion and paste compatibility, offering a novel approach for designing surface-functionalized micron-sized silver powder and developing high-performance electronic pastes.

## 1. Introduction

As photovoltaic power generation rises to prominence as a mainstream renewable energy source in the global energy transition—driven by its vast installation potential and wide applicability—crystalline silicon cell technology, the core enabler of efficient photovoltaic conversion, has become a focal point of both industrial and academic research. This prominence is attributed to its high level of process maturity, stable performance, and strong cost competitiveness. Among various crystalline silicon cell technologies, TOPCon cells have rapidly emerged as the leading solution driving efficiency advancements in the photovoltaic industry, owing to their low contact resistance, high compatibility, and excellent scalability [1,2,3,4,5]. As the core metallization material for TOPCon cells, silver paste plays a decisive role in determining the cell’s photovoltaic conversion efficiency. Within the silver paste system, micron-sized silver powder serves as the conductive phase, constituting 80–90% of the total mass. As the primary functional component, it critically influences the paste’s printability and sintering quality [6,7]. However, the current industrial production of micron-sized silver powder via liquid-phase reduction methods is accompanied by several issues. These include high surface energy, susceptibility to agglomeration, and poor compatibility with organic binders. These issues significantly limit the performance of TOPCon pastes [8,9].

To address the challenges associated with silver powder dispersion and compatibility, surface modification technology has been identified as a promising approach. For example, Deng et al. demonstrated that treating silver powder surfaces with spiral airflow temporarily enhances powder dispersibility [10]. However, after storage, the powder surfaces absorb atmospheric moisture, leading to soft agglomeration and reduced paste compatibility. Tan et al. systematically discussed the surface modification mechanism of oleic acid synergized with mechanical ball milling [11]. The oleic acid coating on the powder surface can significantly improve powder dispersibility and the compatibility between zirconia powder and the binder. However, equipment wear introduces impurities that affect material purity, leading to a decline in material performance [12].

To overcome the drawbacks of dry physical methods, wet chemical modification has gradually become the mainstream approach, offering more stable chemical bonding and precise functionalization [13,14]. Within this domain, fatty acids are frequently employed. Zhou et al. used stearic acid to modify Li_2_CO_3_ powder, effectively improving flowability and increasing the water contact angle to 96.7° [15]. However, the limited grafting density of individual fatty acids creates a bottleneck, restricting further improvements in hydrophobicity. To address this, researchers have explored fatty acid salts. Beretta et al. found that stearates provide superior flowability in dry powders compared to standard stearic acid [16]. Furthermore, Agbaghare et al. demonstrated that the adsorption of fatty acid salts (e.g., sodium palmitate) follows the Freundlich isotherm model, forming a stable, heterogeneous multilayer on powder surfaces [17]. Despite these advantages in molecular-level anchoring and powder dispersion, fatty acid salts feature a single nonpolar carbon chain that exhibits poor compatibility with polar organic carriers. This mismatch frequently causes phase separation or mechanical instability in the prepared slurries, ultimately shortening their shelf life, silane coupling agents are widely regarded as ideal “molecular bridges” that firmly connect inorganic silver powders with organic matrices. Oh et al. showed that KH550 (an aminosilane) generates Si-OH groups through hydrolysis, forming stable covalent bonds with surface hydroxyl groups on powder [18]. Zhang et al. confirmed that these KH550-induced molecular bridges significantly improve the interfacial interaction between micrometer-sized silver flakes and resin matrices, thereby enhancing long-term reliability in humid and hot environments [19]. Yu et al. also successfully optimized the interfacial coupling and dispersion of silver nanowires using KH550 [20]. Highlighting its specific advantages, Xiao et al. compared different silanes and concluded that KH550 provides superior interfacial reinforcement compared to KH560 (which relies on ring-opening reactions) and KH570 (which relies primarily on physical entanglement). This superiority is attributed to the robust network of covalent and hydrogen bonds formed by the terminal active amino groups of KH550 [21].

In summary, fatty acid salt modifiers effectively improve the flow characteristics and primary dispersion of silver powder, whereas silane coupling agents are more effective at bridging the interface between the powder and organic carriers. Therefore, synergistic co-modification with fatty acid salts and silane coupling agents represents a promising route for improving powder dispersion and interfacial compatibility in slurry systems. Beyond the photovoltaic applications, the surface functionalization strategy—particularly the use of KH550 as a “molecular bridge” to enhance inorganic–organic interfacial bonding—holds significant promise for cultural heritage conservation, where the “compatibility principle” (i.e., physicochemical matching between repair materials and original artifacts) is paramount. Meanwhile, silver powder with tailored morphology and dispersibility can serve as a functional filler in conductive adhesives, which are increasingly needed for the restoration of metallic artifacts and precision instrument components that require both mechanical strength and electrical conductivity. The synergistic modification approach developed in this work not only addresses the dispersion and compatibility issues in TOPCon pastes, but also provides a theoretical foundation for designing advanced conservation materials with tunable interfacial properties.

The present study analysed the molecular structural characteristics of KH550 and sodium palmitate and investigated their synergistic modification mechanism on the silver powder surface. Interactions between the modified silver powder and the acrylic resin/solvent system in the organic carrier were further examined. Silver powder dispersibility was evaluated through particle size distribution, particle size span, specific surface area, surface energy, bulk and tapped density, Hausner ratio, and surface hydroxyl content. Paste adaptability was assessed at the paste level through rheological testing (3ITT), line resistivity measurements, and aspect-ratio analysis.

## 2. Materials and Methods

### 2.1. Materials

Experimental reagents: AgNO_3_ (AR ≥ 99.8%, Tongbai Hongxin New Material Co., Ltd., Nanyang, China), L-ascorbic acid (AR ≥ 98.5%, Xilong Chemical Co., Ltd., Shantou, China), Arabic gum (AR ≥ 99.0%, Aladdin’s reagent, Shanghai, China), nitric acid (ω = 65–68%, Tianjin Damao Chemical Reagent Factory, Tianjin, China), ammonia (ω = 25–28%, Tianjin Damao Chemical Reagent Factory, Tianjin, China), sodium palmitate (AR ≥ 98.0%, Aladdin’s reagent, Shanghai, China).

### 2.2. Silver Powder Sample Preparation

**Figure 1:**
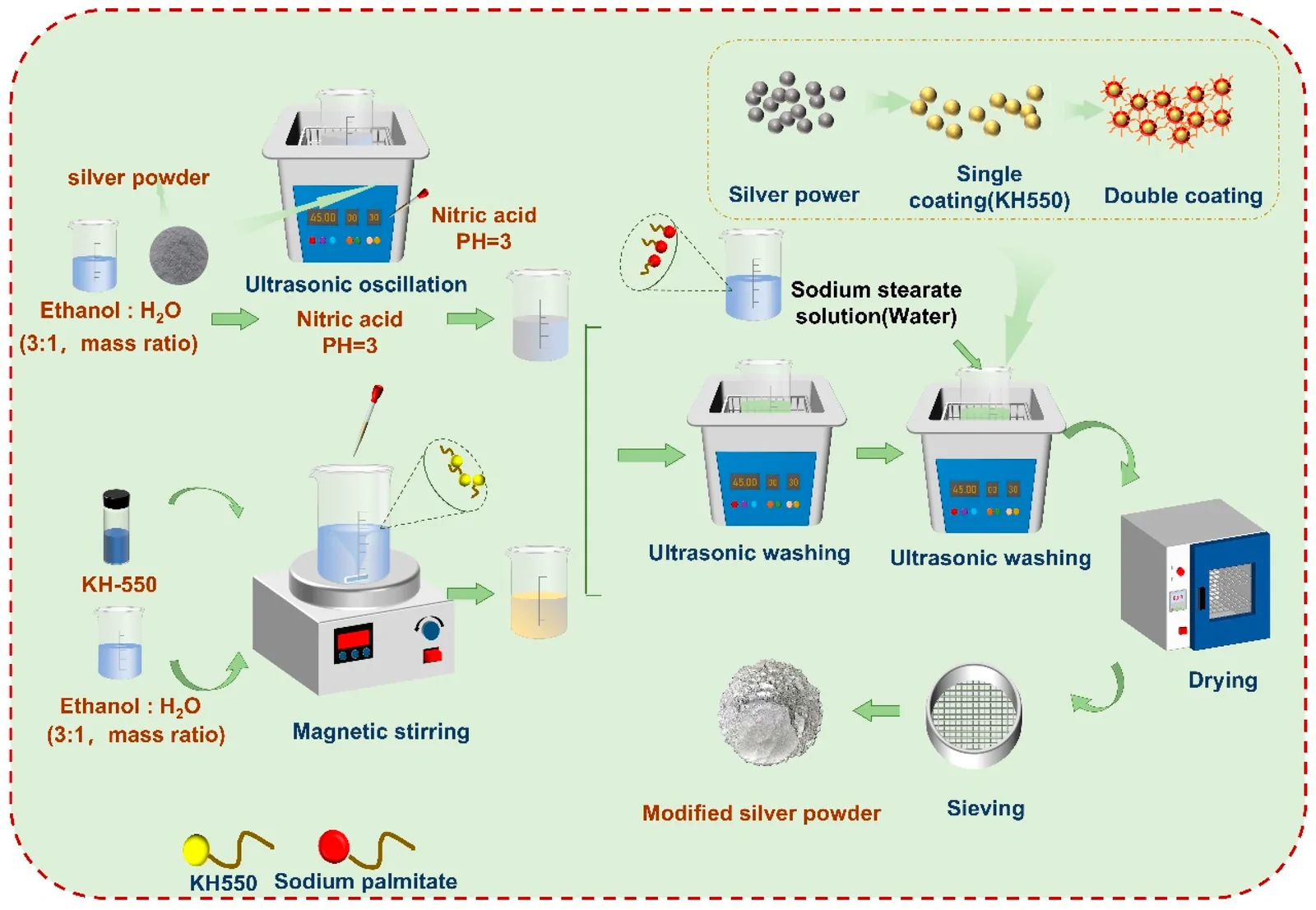
Experimental Flowchart of Silver Powder Composite Modification.

The specific process flow for silver powder composite modification is illustrated in Figure 1. Unmodified silver powder was first dispersed in ethanol and sonicated for 5 min. In parallel, KH550 was added to an ethanol–water mixture with an ethanol/water volume ratio of 3:1, the pH was adjusted to 3, and the solution was hydrolyzed for 30 min. The hydrolyzed KH550 solution was then introduced into the silver powder dispersion, and the mixture was ultrasonicated at 45 °C for 30 min to complete the primary modification. For the Ag@KH550&SP sample, KH550 and sodium palmitate were each added at 0.5 wt% relative to the mass of Ag powder, as listed in Table S2. After the primary modification, sodium palmitate was dissolved in deionized water and added to the modified suspension for secondary modification. The mixture was further ultrasonicated at 45 °C for 30 min, dried at 90 °C for 12 h, ground, and passed through a 450-mesh sieve to obtain the composite-modified silver powder. The liquid-phase reduction procedure used to prepare the micrometer-sized Ag powder is summarized in Table S1.

### 2.3. Characterization of Composite Modified Silver Powder

The BSD-PS-1 Specific Surface Area Analyzer (Beishide Instrument Technology (Beijing) Co., Ltd., Beijing, China) measures the specific surface area of silver powder. The activation temperature was set at 60 °C with an activation duration of 6 h.

The SU-8010 Scanning Electron Microscope (Hitachi, Ltd., Tokyo, Japan) observes the surface morphology of silver powder.

The BT-9300ST Laser Particle Size Analyzer (Dandong Bettersize Instruments Ltd., Dandong, Liaoning, China) tests the D10, D50, and D90 values and their respective percentages for silver powder. Equation (1) defines the particle size span as Span = (D90 − D10)/D50, where D10, D50, and D90 are the particle diameters at 10%, 50%, and 90% cumulative undersize, respectively. A smaller span value indicates a narrower particle size distribution and better size uniformity [22].

(1) $$\mathrm{Span}=\frac{D90-D10}{D50}$$

D8-ADVANCE X-ray diffractometer (Bruker Corporation, Karlsruhe, Germany) studies the phase composition of silver powder. X-ray diffraction (XRD) measurements were performed using Cu Kα radiation (λ = 0.1541 nm). The tests were conducted at an accelerating voltage of 40 kV and a tube current of 40 mA, recorded over a 2θ scanning range of 20–90° at a scan rate of 100°/min.

Bruker Tensor II Fourier Transform Infrared Spectrometer (Bruker Corporation, Germany) was used to investigate the surface chemical bonds of silver powder.

Kratos Axis Ultra X-ray Photoelectron Spectroscopy (XPS) (Kratos Analytical Ltd., Manchester, UK) employed a monochromatic Al Kα source (15 mA, 14 kV), calibrated using the C 1s peak at 284.8 eV, to characterize surface elemental binding in silver powder.

Thermogravimetric analyzer (Q600) (TA Instruments, New Castle, DE, USA) operated under a nitrogen atmosphere with a heating rate of 10 °C/min to 550 °C, analyzing mass loss of silver powder calcined to 500 °C.

A surface profilometer (Bruker DEKTAKXT) (Bruker Nano Surfaces Division, Tucson, AZ, USA) examined the morphology of sintered silver grid lines.

Using the automatic device of the tap density tester (BT-311) (Dandong Bettersize Instruments Ltd., Dandong, Liaoning, China), continuously vibrate 30 g of silver powder for 3000 cycles until a constant volume is achieved, thereby obtaining $\rho_{\mathrm{tb}}$ (tap density). Using the funnel method, allow 30 g of silver powder to fall freely from the funnel opening to a predetermined height until it fills the cup. Take the combined volume of the highest and lowest points of the silver powder as half the loose volume, thereby obtaining $\rho_{\mathrm{lb}}$ (loose bulk density). As shown in Equation (2), the Hausner Ratio (HR) is calculated as the ratio of the tapped density to the bulk density [23]:

(2) $$\mathrm{HR}=\frac{\rho_{\mathrm{tb}}}{\rho_{\mathrm{lb}}}$$

The hydroxyl content of the powder was determined by titration. Briefly, 2 g of sample was dispersed in 250 mL of 20% NaCl solution in a 400 mL beaker, and the pH was adjusted to 4 with 0.1 mol/L HCl. The acid or base used during this pH-adjustment step was not included in the calculation. The suspension was then titrated with 0.1 mol/L NaOH at a rate of 2–3 drops per second until the pH reached 9, and the endpoint was maintained for 5 min. The surface hydroxyl density (n, nm^−2^) was calculated from the measured NaOH consumption (V, mL), the sample specific surface area (S, m^2^/g), and the relationship given in Equation (3).

(3) $$n=\left(\frac{6.02\times 10^{23}}{10^{4}}\times \frac{V}{2}\right)/(S\times 10^{18})$$

A 0.5 g silver powder sample was pressed into a thin sheet and analyzed using a DSA25 contact angle goniometer equipped with a high-speed camera (3000 fps, MotionXtra N3) and a tilting cradle. Contact angle measurements were performed using deionized water, glycerol, and diiodomethane. The surface free energy of the silver powder was calculated using the Owens–Wendt–Rabel–Kaelble (OWRK) method [24].

Equation (4) θ denotes the contact angle. $Y_{L}^{P}{\;\mathrm{and}\;Y}_{L}^{D}$ are the polar and non-polar components of the liquid; $Y_{S}^{P}$ and $Y_{S}^{D}$ represent the polar and non-polar components of the solid, respectively. In Equations (5) and (6), $Y_{L}$ and $Y_{S}$ denote the surface energies of the liquid and solid phases, respectively. The surface energy of silver powder solids was calculated using the simultaneous Equations (4)–(6). The surface energy parameters of the liquids employed in this study are listed in Table S3 [25].

(4) $$Y_{L}\frac{\left(1+\cos\theta \right)}{2{\left(Y_{L}^{D}\right)}^{1/2}}={\left(Y_{S}^{P}\right)}^{1/2}\times {\left(\frac{Y_{L}^{P}}{Y_{L}^{D}}\right)}^{1/2}+{\left(Y_{S}^{D}\right)}^{1/2}$$

(5) $$Y_{L}=Y_{L}^{P}+Y_{L}^{D}$$

(6) $$Y_{S}=Y_{S}^{p}+Y_{S}^{D}$$

### 2.4. Preparation and Printing of TOPCon Slurry

For slurry preparation, the organic carrier components were stirred at 50 °C and 400 rpm for 30 min until fully mixed. Then, 30 g of modified silver powder, 3.79 g of organic carrier, and 0.68 g of glass powder were premixed in a V-type mixer and further dispersed using a three-roll mill until the slurry fineness was below 5 μm. The component ratios of the silver paste are listed in Table S4.

The composition of the TOPCon paste is listed in Table S5. Rheological testing was conducted in rotary mode using an Anton Paar MCR 302 rheometer with a three-stage shear-rate program to compare structural breakdown and recovery among the different paste formulations [26,27,28]. The program consisted of 0.01 s^−1^ for 10 s, 200 s^−1^ for 3 s, and 0.01 s^−1^ for 30 s. This protocol was used as a comparative rheological probe rather than a full reproduction of the instantaneous high-shear conditions experienced during industrial screen-printing squeegee transfer.

For resistivity testing, the fabricated front-side paste was printed onto 20 mm × 20 mm silicon wafers using a 510-mesh screen at a printing speed of 200 mm/s and a pressure of 60 N, forming three grid lines per wafer. Each sample group contained one silicon wafer. After printing, the wafers were dried at 350 °C for 1 min and then sintered three times at 750 °C for 1 min each. After standing at room temperature for 24 h, the resistivity and aspect ratio of the upper, middle, and lower grid lines were measured. Each measurement was repeated three times and the average value was recorded.

In Equation (7), R is the resistance value of the grid line measured by the resistance meter; S is the cross-sectional area of the grid line measured by the surface profilometer; L is the grid line length, 20 mm; and ρ is the resistivity. In Equation (8), Ar is the height-to-width ratio of the grid line cross-section morphology, H is the height of the grid line cross-section, and W is the width of the grid line cross-section.

(7) ρ=RS/L

(8) Ar=H/w

## 3. Results and Discussions

### 3.1. Optimization of Modified Process and Preparation of Silver Powder Modification

As shown in Figure 1, under ultrasonic conditions at 45 °C in deionized water, sodium palmitate (SP) can ionize to release sodium ions and carboxylate ions. The precipitated carboxylate ions primarily interact with the silver powder surface via coordinate bonds, thereby achieving modification [29,30,31]. As a silane coupling agent, KH550 readily undergoes hydrolysis in aqueous solution to form silanols. Excessively rapid hydrolysis rates may lead to excessive condensation polymerization between silanols, resulting in white precipitation.

As shown in Figure S1, the hydrolysis and condensation behavior of KH550 was strongly affected by both the solvent composition and the pH [32,33,34]. In pure water (WH), the conductivity changed sharply, indicating rapid hydrolysis accompanied by rapid condensation. In pure ethanol (EH), hydrolysis was strongly suppressed because ethanol stabilized the ethoxy groups of KH550. In the mixed solvent system EWH (ethanol:water:KH550 = 36:12:1), the conductivity evolution became more stable after approximately 30 min, indicating a more controllable hydrolysis process. The pH also played a critical role. At pH = 1 (Figure S1a), strong protonation was unfavorable for the subsequent interaction between KH550 and sodium palmitate. At pH = 7 (Figure S1c), the conductivity decreased to below 100 μS/cm after 30 min, suggesting insufficient hydrolysis activity for efficient surface grafting. By contrast, at pH = 3 (Figure S1b), the mixed solvent system reached a relatively stable conductivity after 30 min and was therefore selected as the optimal hydrolysis condition for subsequent modification. Accordingly, an ethanol–water mixed solvent with a volume ratio of 3:1, adjusted to pH 3 and hydrolyzed for 30 min, was used throughout this study.

Due to the use of a liquid-phase reduction process, the silver powder prepared in this study is naturally coated with a large number of active hydroxyl groups on its surface. Based on this interfacial characteristic, the silanol groups of the modified molecules undergo dehydration condensation with the surface hydroxyl groups, forming stable Ag-O-Si covalent bonds; simultaneously, the amino groups at the distal ends of the molecules coordinate with silver atoms, thereby achieving a synergistic adsorption effect.

**Figure 2:**
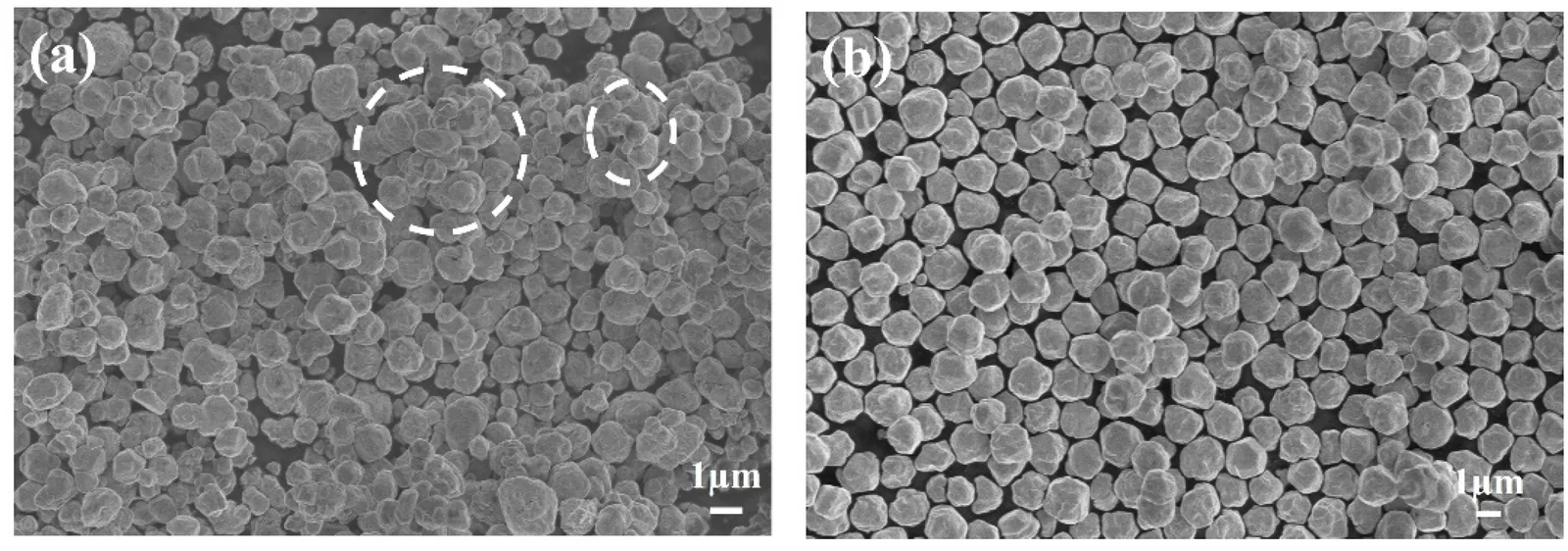
SEM images of silver powder: (**a**) before modification (the dashed circles denote typical agglomerated regions); (**b**) after modification.

Figure 2a shows that the unmodified silver powder contained particles with different degrees of agglomeration, including both soft and hard agglomerates [35,36]. After composite modification, the extent of soft agglomeration was clearly reduced and the dispersion state was improved (Figure 2b). The bottle-wall adhesion test shown in Figure S2 was used only as a qualitative comparison of the wetting behavior of the powders under the same conditions. The unmodified powder showed weak adhesion to the glass wall, whereas the modified powder adhered more strongly. This observation, together with the contact-angle and surface-energy results discussed below, is consistent with a change in surface wettability after modification, although the bottle-wall test alone does not directly prove high surface energy or hydrophilicity [37,38].

### 3.2. Characterisation of Silver Powder Coating

The sample labels used in this study are defined as follows: SP, sodium palmitate; Ag, raw silver powder; Ag@SP, SP-modified silver powder; Ag@KH550, KH550-modified silver powder; Ag@KH550&SP, silver powder modified sequentially with KH550 followed by SP; Ag@(SP+KH550), silver powder modified with SP and KH550 simultaneously; and Ag@SP&KH550, silver powder modified sequentially with SP followed by KH550. Specific formulation parameters are provided in Table S2.

**Figure 3:**
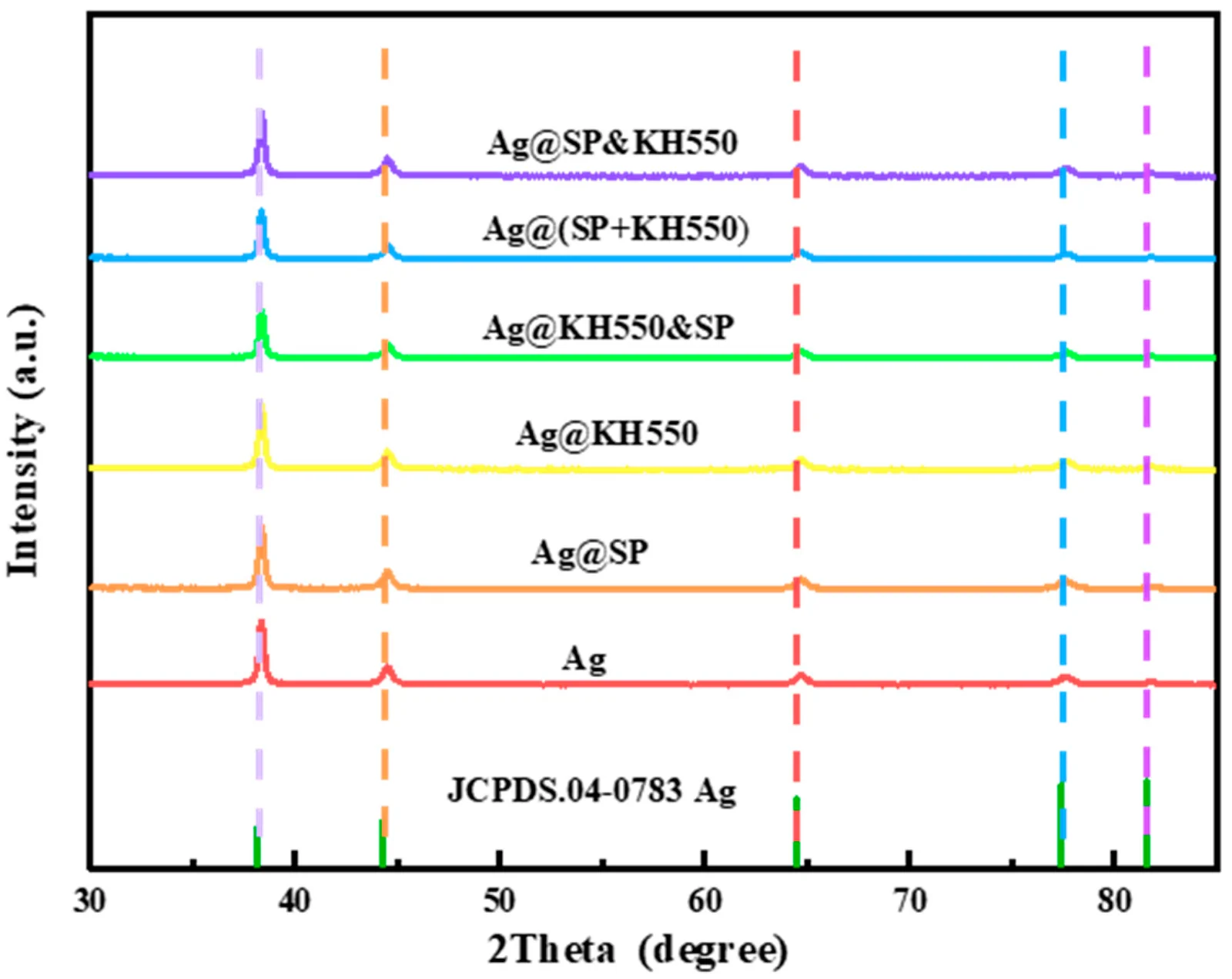
XRD of different samples.

According to the XRD in Figure 3, the diffraction pattern of sample Ag exhibits intense peaks at 38.11°, 44.29°, 64.44°, 77.39°, and 81.53°. These peaks precisely correspond to the (111), (200), (220), (311), and (222) crystal planes of face-centered cubic (fcc) silver (consistent with JCPDS card 04-0783). No characteristic peaks from other crystalline phases were detected, and the narrow half-widths of the silver diffraction peaks clearly indicate the excellent purity and high crystallinity of the prepared silver powder. Comparative analysis of the XRD patterns for a series of modified samples—Ag@SP, Ag@KH550, Ag@KH550&SP, Ag@(SP+KH550), and Ag@SP&KH550—observed that the positions of all characteristic peaks remained unchanged (i.e., the lattice constant remained constant). Furthermore, neither the full width at half maximum (FWHM) nor the relative peak intensity of the diffraction peaks exhibited significant alterations. This conclusively demonstrates that the adsorption of the modifier onto the silver powder surface constitutes a surface modification process, exerting no influence on the lattice parameters or internal microstructure of the silver powder.

**Figure 4:**
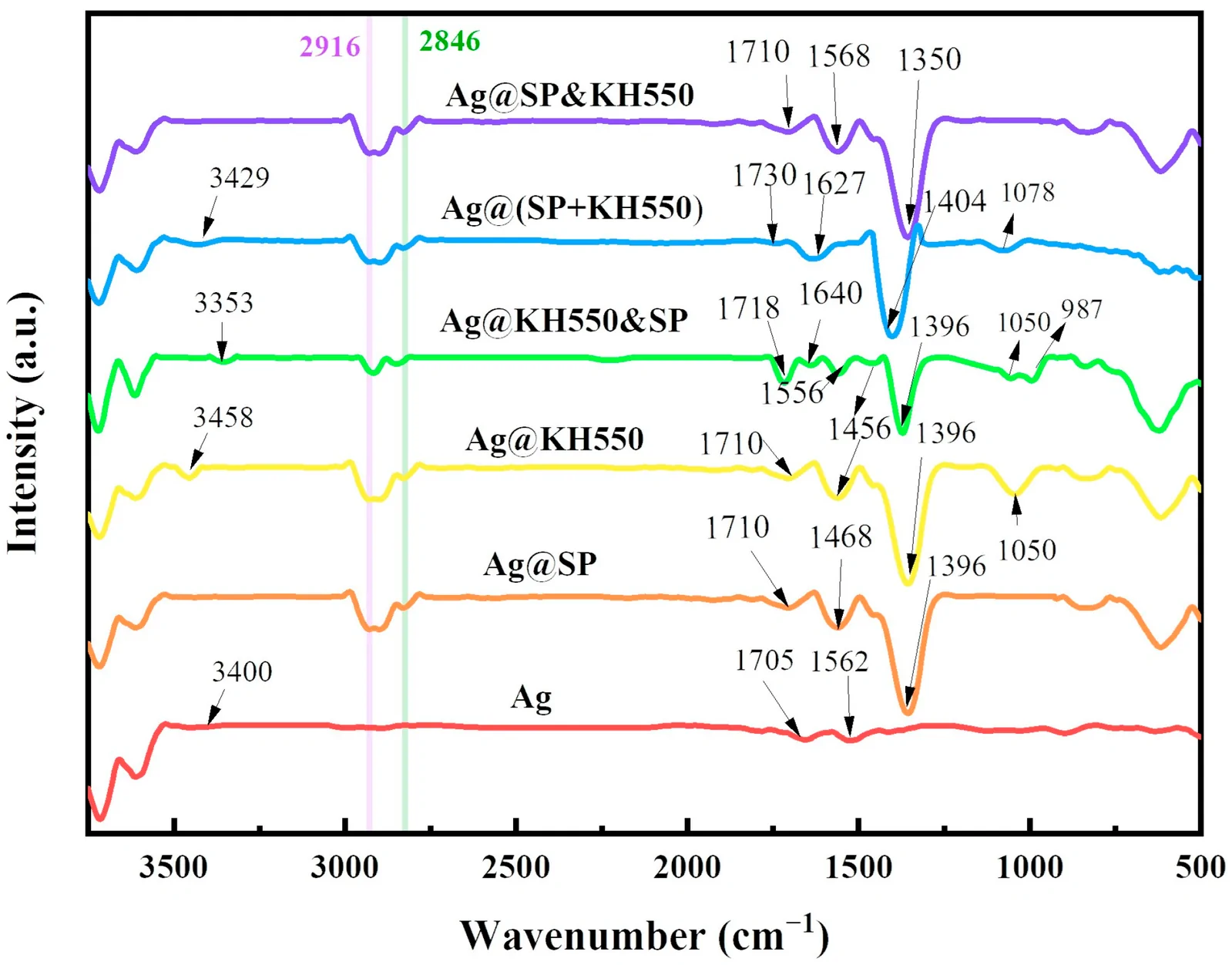
FT-IR of different samples.

The FT-IR spectrum in Figure 4 reveals that the Ag@SP sample exhibits a C=O stretching vibration peak at 1710 cm^−1^ for -COO^−^, attributable to C_15_H_31_COO^−^. Concurrently, bending vibration peaks for -CH_3_ appear at 1468 cm^−1^ and 1396 cm^−1^, while peaks at 2916 cm^−1^ and 2846 cm^−1^ correspond to the asymmetric and symmetric C-H stretching vibrations of the -CH_2_-, respectively, accompanied by Si-OH bond signals. This indicates successful grafting of the C_15_H_31_COO^−^ modification layer onto the sample surface. The Ag@KH550 sample exhibits an N-H stretching vibration peak near 3458 cm^−1^ and -CH_3_ bending vibrations at 1456 cm^−1^ and 1396 cm^−1^, while a characteristic Si-O-Si bond peak formed at 1010 cm^−1^, confirming surface modification with KH550. The Ag@KH550&SP sample exhibits an overlapping peak at 3353 cm^−1^ corresponding to N-H stretching vibrations and Si-OH bonds, 2916 cm^−1^ and 2846 cm^−1^ correspond to methylene C-H stretching vibrations, 1718 cm^−1^ is attributed to the C=O vibration of C_15_H_31_COO^−^, 1640 cm^−1^ and 1556 cm^−1^ represent the C=O stretching vibration of the amide bond -CONH- group, while 1456 cm^−1^ and 1390 cm^−1^ correspond to -CH_3_ bending vibrations. The peaks at 1050 cm^−1^ and 987 cm^−1^ correspond to Si-O-Si stretching vibrations. These results indicate the formation of an amide bond between C_15_H_31_COO^−^ and the -NH_2_ group of KH550, confirming the simultaneous introduction of KH550 and C_15_H_31_COO^−^ onto the silver powder surface and the formation of a chemical bond. In contrast, the Ag@(SP+KH550) sample exhibited only the C=O vibration peak of C_15_H_31_COO^−^ at 1730 cm^−1^, alongside a composite peak at 3429 cm^−1^ for N-H and Si-OH bonds, with the remaining characteristic peaks of the KH550 structure conspicuously absent; in the Ag@SP&KH550 sample, the peaks at 1710 cm^−1^ and 1568 cm^−1^ correspond to the C=O vibration and the asymmetric stretching vibration of the -COO^−^ group in C_15_H_31_COO^−^, respectively. The attenuated intensity or absence of characteristic peaks in the FT-IR spectra of Ag@(SP+KH550) and Ag@SP&KH550 suggests that a suboptimal addition sequence of the modifiers hindered effective adsorption. This led to deficient interfacial bonding and discontinuous encapsulation, underscoring the pivotal role of the procedural sequence in governing surface modification efficacy.

**Figure 5:**
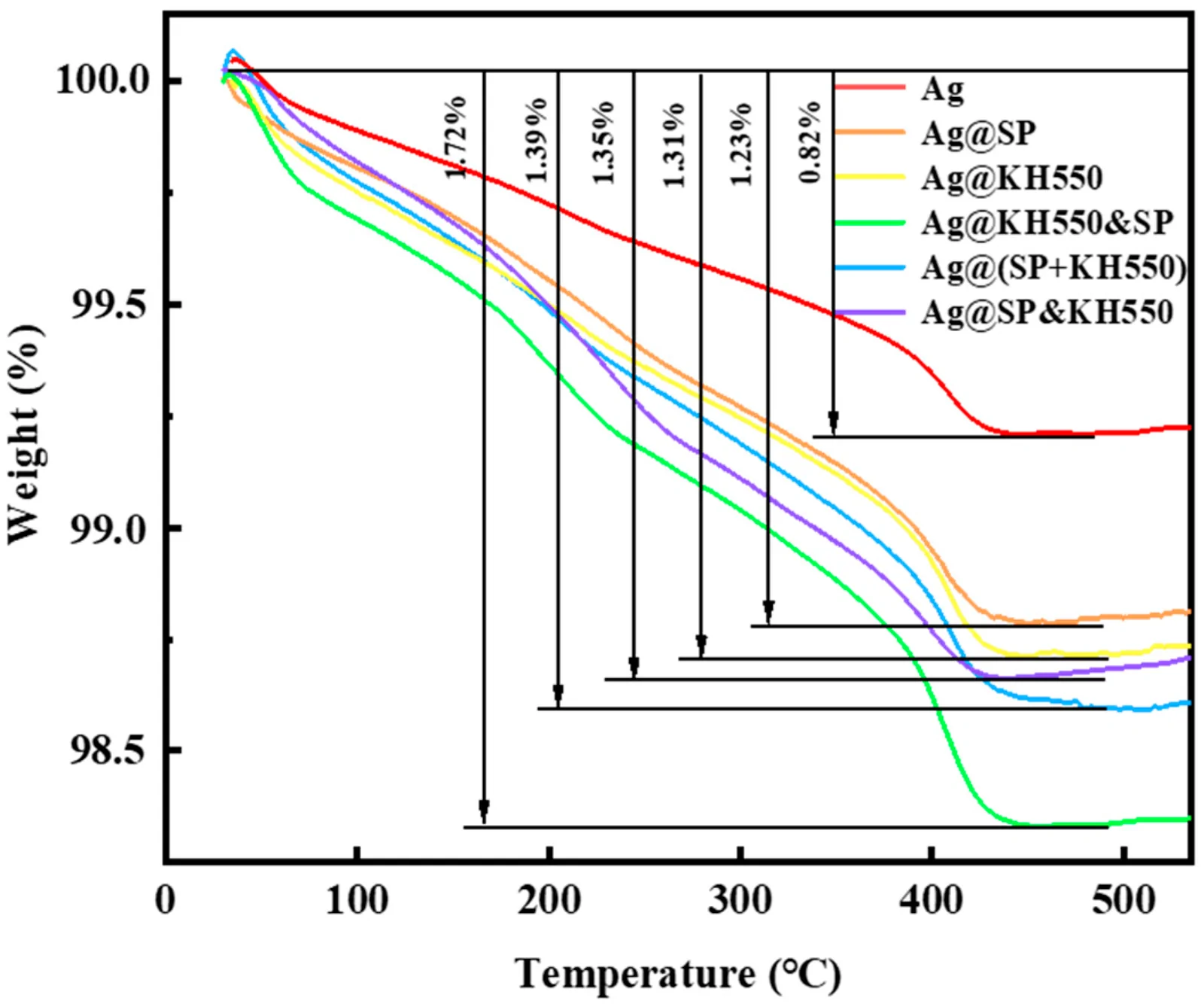
TGA curve of different samples.

The thermogravimetric (TGA) curve shown in Figure 5 indicates that the Ag sample exhibits a mass loss of 0.82% at 500 °C (m_0_), primarily attributable to the volatilization of physically adsorbed moisture and residual dispersant. In contrast, the Ag@SP and Ag@KH550 samples treated with single modifiers both exhibited losses below 1.32%, confirming the lower grafting efficiency of single modification (whether through physical adsorption or limited chemical adsorption).

The Δm values of the composite-modified samples Ag@KH550&SP, Ag@(SP+KH550), and Ag@SP&KH550 all showed improvement, providing strong evidence that the composite strategy of KH550 and SP significantly enhances the grafting rate of the modifier (Δm values refer to the net additional mass loss relative to the unmodified Ag baseline). On this basis, Ag@KH550&SP exhibited the largest net organic contribution (Δm = 0.90%) among the composite-modified samples, whereas Ag@(SP+KH550) and Ag@SP&KH550 showed lower values of 0.57% and 0.53%, respectively. These results suggest that the sequential KH550-then-SP route produced the highest effective organic loading on the Ag surface.

**Figure 6:**
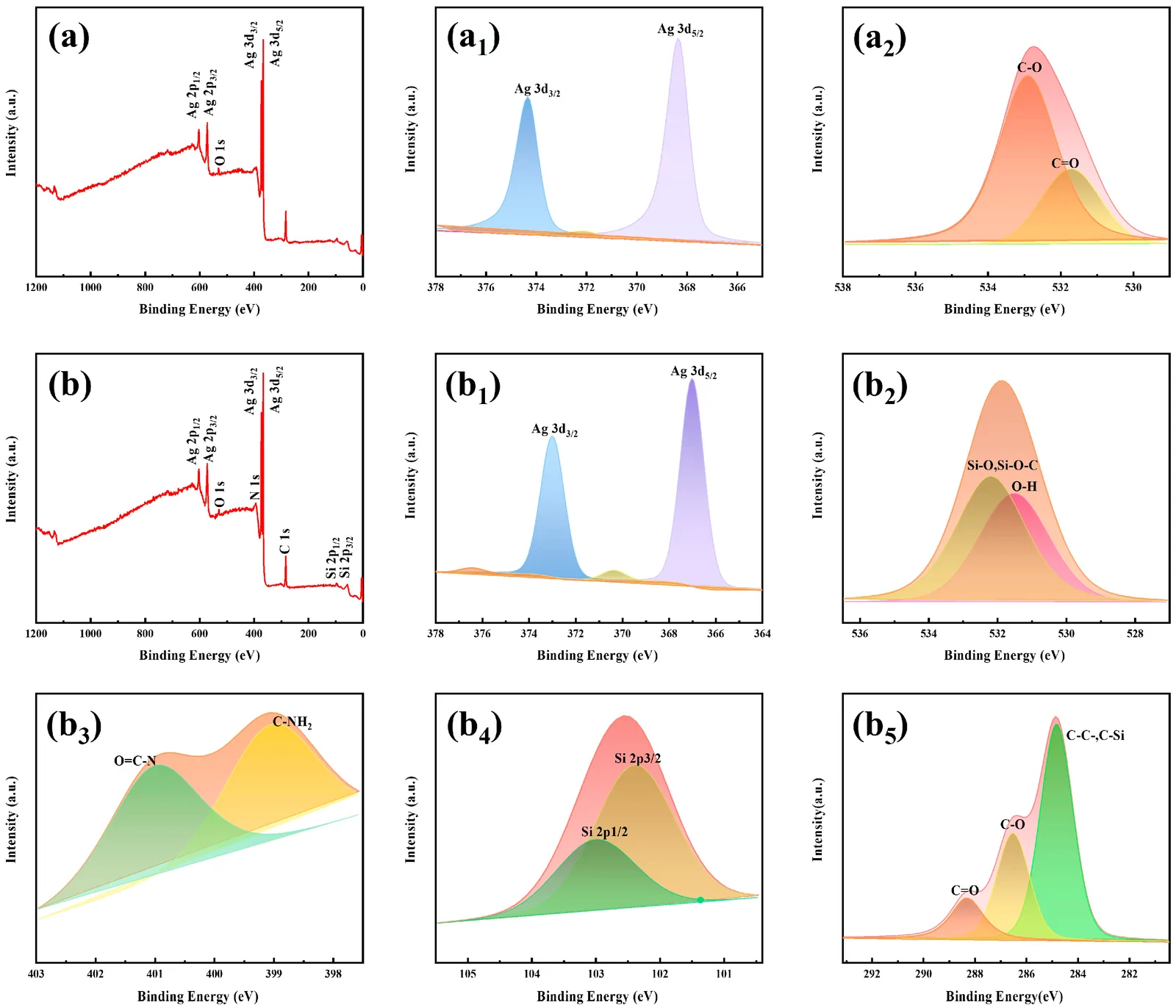
The XPS spectra of Ag (**a**–**a_2_**) and Ag@KH550&SP (**b**–**b_5_**).

The surface elemental composition and chemical bonding states of the synergistically modified silver powder are shown in Figure 6a,b. No Na-related peak was detected in the survey spectrum, indicating that Na+ released from SP dissociation in the solvent was not retained on the Ag powder surface after modification. In the Ag 3d spectra (Figure 6a_1_,b_1_), the characteristic Ag 3d5/2 and Ag 3d3/2 peaks were clearly observed, together with the corresponding Ag energy-loss feature. The O 1s spectrum (Figure 6b_2_) showed a component at 532.18 eV, which was assigned to Si-O and Si-O-Si species generated by the hydrolysis and condensation of KH550, as well as a component at 531.5 eV attributable to surface hydroxyl groups derived from the Ag powder and residual Si-OH species of KH550. The Si 2p spectrum (Figure 6b_4_) displayed the characteristic Si 2p1/2 and Si 2p3/2 doublet of organosilicon species, confirming that KH550 was chemically introduced onto the Ag surface. More importantly, quantitative deconvolution of the high-resolution C 1s spectrum yielded three components at 284.8 eV (FWHM = 1.41 eV, 50.97 at.%), 286.5 eV (FWHM = 1.80 eV, 35.89 at.%), and 288.0 eV (FWHM = 1.80 eV, 13.14 at.%), which were assigned to C-C/C-Si, C-O, and carbonyl-containing species, respectively. The N 1s spectrum was fitted with peaks at 399.04 eV (FWHM = 2.92 eV, 32.35 at.%) and 401.09 eV (FWHM = 4.96 eV, 67.65 at.%), corresponding to C-NH_2_ and O=C-N-related nitrogen environments, respectively. The presence of the carbonyl-containing C 1s component at 288.0 eV, together with the O=C-N-related N 1s component at 401.09 eV, confirms that amidation occurred after sequential KH550/SP treatment. However, the carbonyl contribution accounts for only 13.14 at.% of the fitted C 1s envelope, whereas the higher-binding-energy N component accounts for 67.65 at.% of the fitted N 1s envelope. This result suggests that only part of the high-binding-energy nitrogen originates from amide formation, while a substantial fraction is more reasonably attributed to strongly bound interfacial nitrogen associated with protonated amine–carboxylate interactions. Therefore, the XPS results indicate a surface bonding process in which covalent Ag–O–Si anchoring is accompanied by both ionic interactions and partial amidation, with ionic interactions likely playing a larger role in the secondary contribution.

### 3.3. Physical Testing of Silver Powder

**Figure 7:**
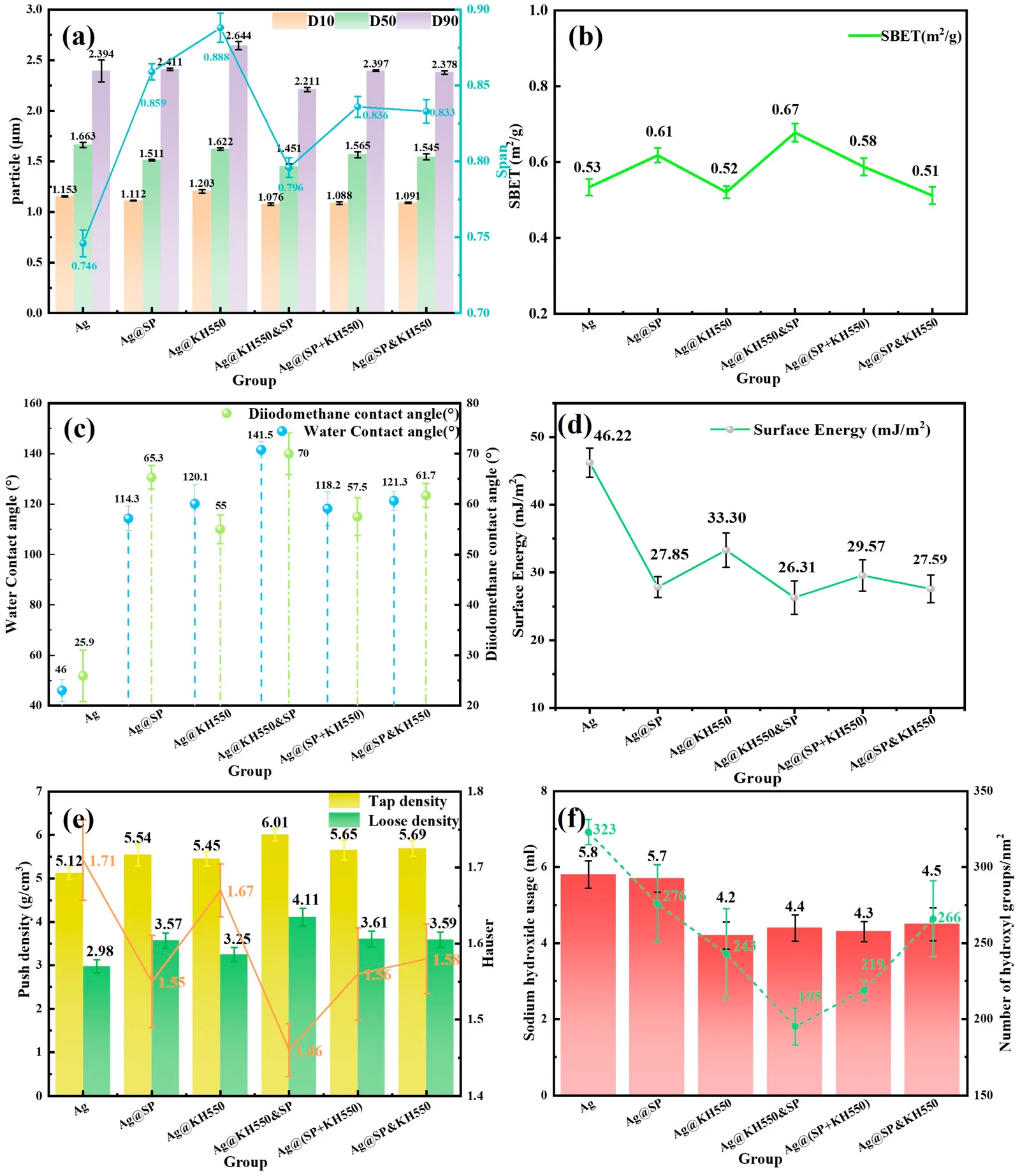
Physical property testing of different samples: (**a**) particle size and span, (**b**) specific surface area, (**c**) contact angles with water and diiodomethane, (**d**) surface energy, (**e**) tapped and bulk density and Hauser, (**f**) volume of NaOH solution consumed per nm^2^ of hydroxyl groups on the silver powder surface.

The Ag@KH550&SP sample achieved the smallest D10, D50, and D90 values, with D50 as low as 1.451 μm, confirming the synergistic effect of composite modification (Figure 7a). As shown in Figure 7b, the Ag@SP, Ag@(SP+KH550) and Ag@KH550&SP samples display elevated SBET values, which evidences partial deagglomeration of silver particle agglomerates. This leads to reduced overall particle size and improved dispersion. Figure 7c,d indicate that the Ag sample exhibits hydrophilicity due to its high surface hydroxyl content (water contact angle of 46°, surface energy of 37.56 mJ/m^2^). After SP modification, the encapsulation of nonpolar long carbon chains transforms the silver powder surface into a hydrophobic state (water contact angle of 114.3°), with a significant reduction in surface energy (27.85 mJ/m^2^). KH550 modification utilizes adsorption between Si-OH and -OH groups alongside Si-O-Si bond formation. The Ag@KH550&SP sample employs a composite strategy of KH550 followed by SP, where KH550 first forms a stable chemisorbed layer on the silver powder surface. Subsequently, SP (C_15_H_31_COO^−^) is directionally grafted onto KH550’s active sites (-NH_3_^+^) via strong ionic bonding forces or covalent bonds, constructing a dense hydrophobic barrier that significantly enhances coating efficiency and modification effectiveness. In contrast, the Ag@(SP+KH550) and Ag@SP&KH550 samples exhibit limited performance improvement due to incomplete interfacial bonding caused by the improper adsorption sequence of the modifier. Figure 7e indicates that both the tapped density and bulk density of the modified silver powder increased, with improved flowability. The Ag@KH550&SP sample exhibited optimal packing properties (tapped density: 6.01 g/cm^3^, bulk density: 4.11 g/cm^3^) and the lowest Hausner ratio (1.46), indicating the weakest interparticle forces and optimal dispersion. This result validates the synergistic mechanism where KH550 provides steric hindrance while SP chains suppress van der Waals forces, effectively inhibiting agglomeration and enhancing the stress transfer capability of the silver powder. Figure 7f shows the NaOH titration volume and reveals the performance differences among various modification strategies. The Ag sample exhibits a high surface hydroxyl density (323/nm^2^), indicating high surface energy, numerous active sites, and hydrophilic characteristics. In contrast, the surface hydroxyl density of the Ag@KH550&SP sample is significantly reduced to 195/nm^2^, demonstrating effective suppression of surface energy and overall nonpolar characteristics of the material. The substantial decrease in hydroxyl content confirms KH550’s pivotal role as a “molecular bridge”: its siloxane end undergoes condensation reactions with silver powder surface hydroxyls, consuming hydroxyl groups while constructing a reactive interface terminated with amino groups. This interface provides grafting sites for SP’s carboxyl groups. Through partial amidation reactions, they form robust chemical bonds and potentially partial ionic bonds, collectively constituting a stable organic coating layer. This synergistic mechanism not only efficiently eliminates surface hydroxyls but also fundamentally transforms the interface properties, significantly enhancing its hydrophobicity and compatibility with organic phases.

In summary, the sequential composite modification of KH550 and SP demonstrates significant synergistic enhancement effects in reducing particle size, lowering surface energy, and improving the flowability and bulk density of silver powder by constructing a stable interfacial layer and hydrophobic barrier. Based on the characterization results in Figure 7, composite modifiers demonstrate greater effectiveness than single modifiers in reducing powder particle size and improving dispersion. Among these, the Ag@KH550&SP sample exhibits the most favorable overall performance. Subsequent investigations into the surface characteristics of silver powder and slurry properties will focus on the Ag@KH550&SP, Ag@(SP+KH550), and Ag@SP&KH550 samples.

### 3.4. TOPCon Paste Performance

**Figure 8:**
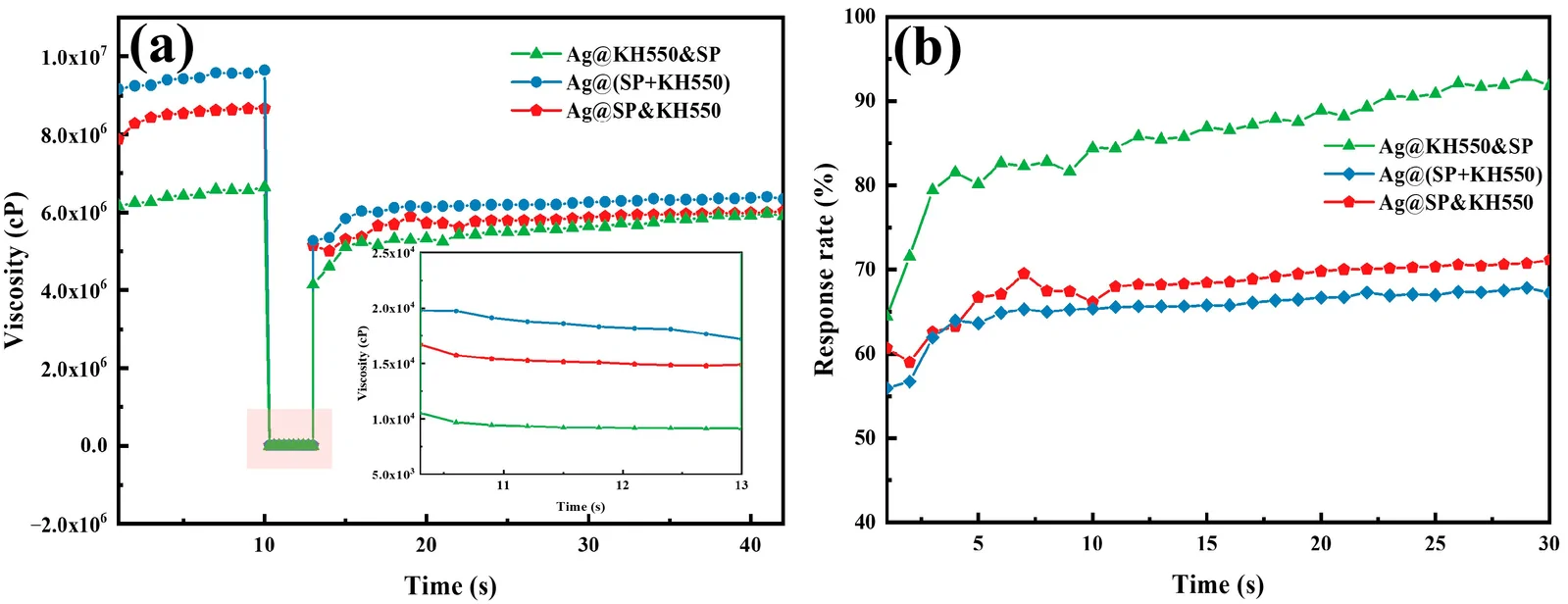
3ITT (**a**) and recovery rate (**b**) of Ag@KH550&SP, Ag@(SP+KH550), and Ag@SP&KH550.

The Ag@KH550&SP sample exhibited the most favorable rheological properties (Figure 8a,b). Its first-stage average viscosity was 6.43 × 10^6^ cP, which was lower than those of the Ag@SP and Ag@KH550 samples (>10^7^ cP), indicating that the composite modification improved the dispersion state of the silver powder in the paste system. In addition, Ag@KH550&SP achieved a structural recovery rate of 88.3% within 17 s, which was markedly higher than those of Ag@(SP+KH550) (65.7%) and Ag@SP&KH550 (68.9%). This behavior is consistent with stronger interfacial interactions between the modified particle surface and the organic-carrier matrix, which promote rapid reconstruction of the paste network after shear [39,40,41]. Because the maximum shear rate used in the 3ITT test was substantially lower than the instantaneous shear rate experienced during industrial squeegee transfer, the 3ITT results are interpreted here as comparative indicators of flow and recovery behavior rather than a complete simulation of industrial printing dynamics.

Additionally, the Ag@KH550&SP sample showed pronounced shear-thinning behavior in the second stage, suggesting that the modified particles aligned more readily along the flow direction and thereby reduced flow resistance [42,43]. Together with the faster structural recovery, this result indicates that the Ag@KH550&SP paste possessed the best process adaptability for screen-printing applications among the tested samples.

**Figure 9:**
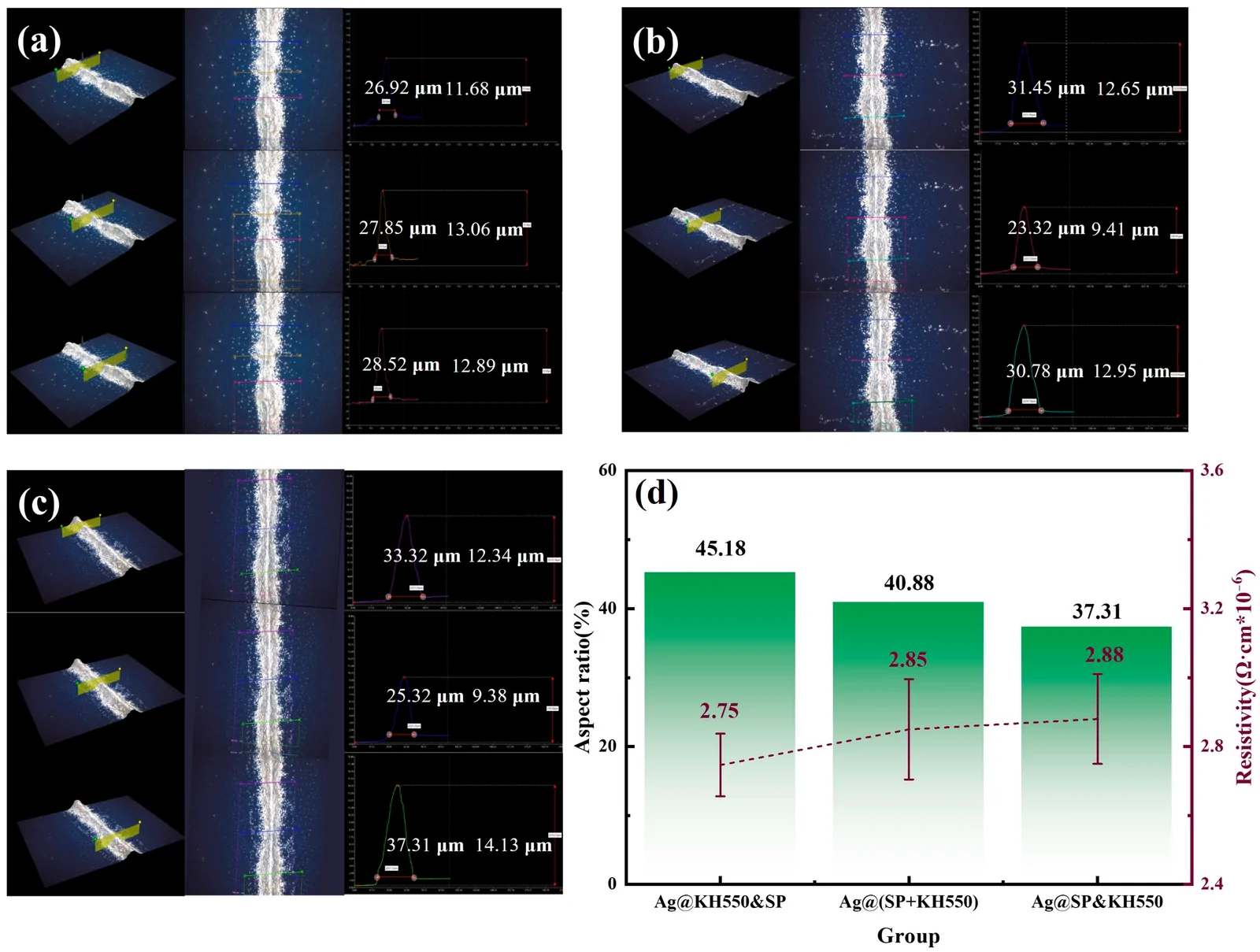
Surface profile (**a**–**c**) and aspect ratio and body electrical resistivity (**d**) of Ag@KH550&SP, Ag@(SP+KH550), and Ag@SP&KH550.

The Ag@KH550&SP sample exhibits the optimal aspect ratio of the formed grid lines, reaching 45.18% (Figure 9a–c). This represents an improvement of 4.3% and 7.87% compared to the Ag@(SP+KH550) sample (40.88%) and the Ag@SP&KH550 sample (37.31%), respectively. The higher aspect ratio indicates the slurry possesses excellent thixotropy and structural recovery capability, facilitating high conductivity and low light-blocking loss [44,45,46]. In terms of electrical performance, the resistivity of the Ag@KH550&SP sample in Figure 9d is 2.75 × 10^−6^ Ω·cm. This value is within the same order of magnitude as those measured for the Ag@(SP+KH550) and Ag@SP&KH550 samples. This suggests that, at a sintering temperature of 750 °C, the effect of the modification treatment on the resistance of the silver powder is reduced due to the high temperature. This indicates that under the sintering condition of 750 °C, the high temperature weakens the regulatory effect of the modification treatment on the silver powder resistance. At this temperature, the modifier is essentially fully volatilized, and the interfacial advantages conferred by functional groups on the silver powder surface are also relatively diminished. High temperatures promote the complete melting and densification of the silver powder layer, bridging the microstructural differences introduced by different post-treatment methods. This results in all samples approaching a fully dense state, thereby diminishing the influence of the silver powder’s initial surface properties on the final resistance [47]. In addition, decomposition of the organic shell during fast co-firing may influence glass-frit wetting and etching, silver crystallite growth at the silicon interface, and possible carbonaceous residue at the contact region, although these interface-level effects were not directly resolved in the present study.

### 3.5. Structure–Activity Relationship Analysis of KH550 and Sodium Palmitate

**Figure 10:**
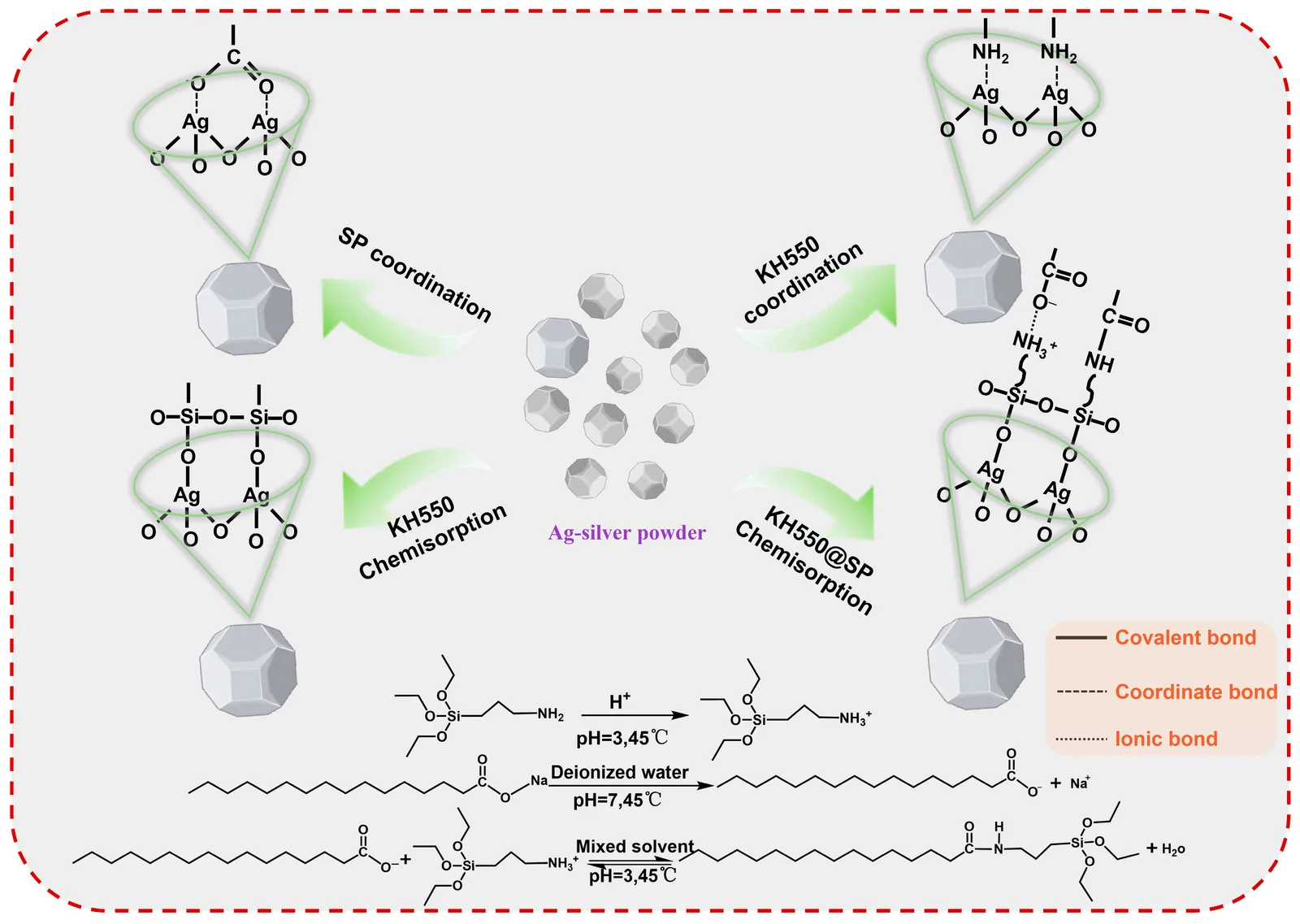
Schematic Diagram of Silver Powder Interface Bonding.

The proposed stepwise modification strategy is illustrated in Figure 10. After hydrolysis, the Si-OH groups of KH550 can react with hydroxyl groups on the silver powder surface to form Ag-O-Si bonds, while the terminal amino group remains available for subsequent interfacial interaction. After ionization of SP, the carboxylate group can interact with Ag or with protonated amino groups derived from KH550. Under the selected acidic conditions (pH = 3), KH550 hydrolysis proceeds in a relatively controlled manner, which facilitates its adsorption onto the silver powder surface. Subsequent addition of SP can then enhance surface coverage through ionic interaction and possible partial amidation under ultrasonication and heating. This sequential process is consistent with the observed reduction in surface energy and the improved dispersibility of the modified silver powder.

## 4. Conclusions

Based on a composite modification system combining the silane coupling agent KH550 and sodium palmitate (SP), this study prepared surface-modified silver powder with reduced surface energy and improved packing behavior, and further evaluated its application in TOPCon photovoltaic silver paste. The results suggest that sequential KH550 and SP treatment alters the surface chemistry of the silver powder through a combination of silane grafting and subsequent interfacial association with SP, thereby improving dispersion and compatibility with the organic carrier. Quantitatively, the modified powder exhibited a tapped density of 6.01 g/cm^3^, a bulk density of 4.11 g/cm^3^, and a markedly reduced surface hydroxyl density. The corresponding paste showed a viscosity of 6.43 × 10^6^ cP, a resistivity of 2.75 × 10^−6^ Ω·cm after sintering, and a printed-line aspect ratio of 45.18%. However, further investigation is still required to determine whether this modification contributes to other performance improvements, such as the storage stability of the paste, and to elucidate the complete mechanism of performance enhancement through contact resistance and photovoltaic current–voltage parameters. Overall, the composite modification strategy improved the dispersion behavior and process adaptability of silver powder in the paste system.

## Data Availability

The original contributions presented in this study are included in the article/Supplementary Material. Further inquiries can be directed to the corresponding author.

## Supplementary Materials

The following supporting information can be downloaded at https://www.mdpi.com/article/10.3390/ma19183939/s1, Table S1: Preparation of silver powder; Table S2: Composition of modified silver powder; Table S3: Surface free energy parameters of liquids used for contact angle measurements; Table S4: Slurry component proportions; Table S5: Composition of slurry and its proportions; Figure S1: Conductivity of KH550 in WH (water:KH550 = 12:1), EH (ethanol:KH550 = 36:1), and EWH (ethanol:water:KH550 = 36:12:1) at (a) pH = 1, (b) pH = 3, and (c) pH = 7; Figure S2: Bottle-wall adhesion test of (a) unmodified silver powder and (b) modified silver powder.
