# Supplementary material for: KH550 and Sodium Palmitate Mediated Synergistic Modification of Micronized Silver Powder: Insights into Dispersion Regulation and TOPCon Paste Performance Optimization
Source: Materials (Basel). 2026 Sep 16;19(18):3939. doi: 10.3390/ma19183939 (PMC13608602; doi:10.3390/ma19183939)
Supplement: Supplementary file 1 [file materials-19-03939-s001.zip › materials-4530355-supplementary.pdf]

Supporting Information

KH550 and Sodium Palmitate Mediated Synergistic Modification of Micronized Silver Powder: Insights into Dispersion Regulation and TOPCon Paste Performance Optimization

Jun Yin [1], Ruxiao Jia [1], Kejun Zhong [1], Lei Zhang [2], Jia Liu [1], Lianzhou Zhang [1], Hao Sun [3], Wenyan Zhang [1], Ying Zhao [4] and Mi Zhang [5,*]

1 Key Laboratory of Synthetic and Natural Functional Molecule of the Ministry of Education, College of Chemistry & Materials Science, Northwest University, Xi'an 710127, China
2 Emperor Qinshihuang's Mausoleum Site Museum, Xi'an 710600, China
3 Xi'an Huichuang Precious Metal New Materials Research Institute Co., Ltd., Xi'an 710300, China
4 Xi'an Hongxing Electronic Paste Technology Co., Ltd., Xi'an 710199, China
5 Shaanxi Provincial Institute of Basic Sciences (Chemistry & Biology), Northwest University, Xi'an 710127, China
* Correspondence: 18192132271@163.com

Table S1. Preparation of Silver Powder.

Condition Name	Condition
Silver nitrate	1mol/L(8mlNitric acid) 510g
Ascorbic acid	0.6 mol/L
Dispersant	Gum Arabic 5%(MW=40000)
Rotational speed	500rpm
Temperature	25℃
Flow rate	60ml/min
Substrate solution	Base solution: 6L of water (7mL of nitric acid)

Table S2. Composition of Modified Silver Powder.

No	Ag/g	Ag@SP -wt%	Ag@KH55 0 -wt%	Ag@KH550 &SP-wt%	Ag@(SP+KH5 50)-wt%	Ag@SP&KH55 0-wt%
1	30	0	0	0	0	0
2	30	1	0	0	0	0
3	30	0	1	0	0	0
4	30	0	0	0.5-0.5	0	0
5	30	0	0	0	0.5-0.5	0
6	30	0	0	0	0	0.5-0.5

Table S3. Surface free energy parameters of liquids used for contact angle measurements.

Solvent	Surface energy (mJ/m^2)		
	γ_L	γ_L^P	γ_L^D
Water	72.8	51.0	21.8
Glycerol	64.0	30.0	34.0
Diiodomethane	50.8	0	50.8

Table S4. Slurry Component Proportions.

Sample	Ag powder	Organic carrier	Glass powder
Slurry mass fraction (wt%)	89	9	2

Table S5. Composition of Slurry and Its Proportions.

	Organic carrier				Solid components
Slurry formulation	Thixotropic agent and Binder	Solvent	Curing agent and Additive	Resin	powder
Quality proportion (wt%)		9			91

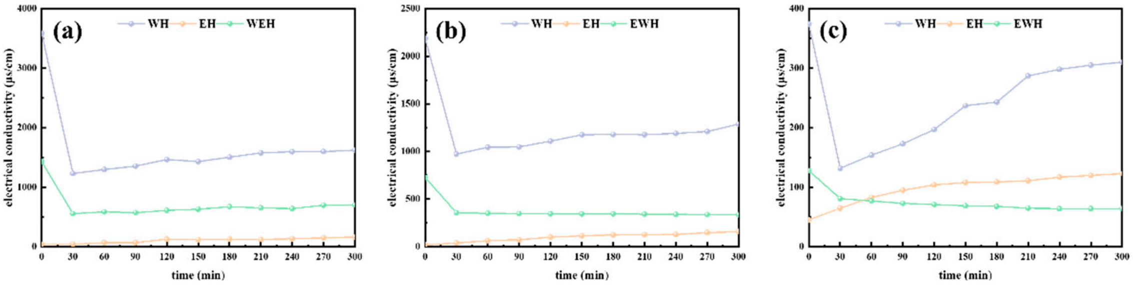

Figure S1. Conductivity of KH550 in WH (water:KH550 = 12:1), EH (ethanol:KH550 = 36:1), and EWH (ethanol:water:KH550 = 36:12:1) at (a) pH = 1, (b) pH = 3, and (c) pH = 7

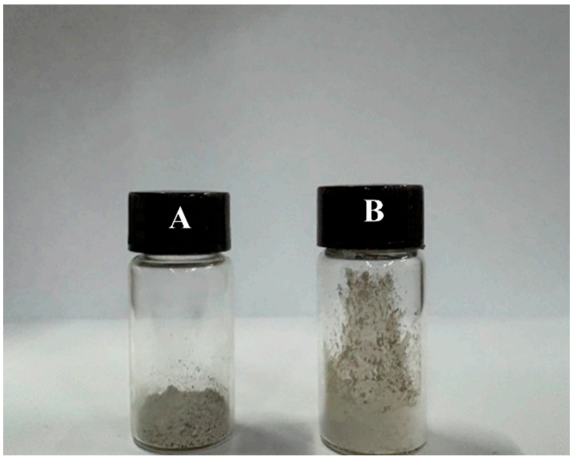

Figure S2. Bottle-wall adhesion test of (a) unmodified silver powder and (b) modified silver powder.
